# Complete genome sequence of the OXA-48-producing *Klebsiella pneumoniae* strain 11978

**DOI:** 10.1128/mra.00341-24

**Published:** 2024-08-20

**Authors:** Otávio Hallal Ferreira Raro, Patrice Nordmann, Laurent Poirel

**Affiliations:** 1 Medical and Molecular Microbiology, Faculty of Science and Medicine, University of Fribourg, Fribourg, Switzerland; 2 Swiss National Reference Center for Emerging Antibiotic Resistance (NARA), University of Fribourg, Fribourg, Switzerland; University of Maryland School of Medicine, Baltimore, Maryland, USA

**Keywords:** *Klebsiella pneumoniae*, Illumina sequencing, Nanopore sequencing, *bla*_OXA-48_ gene

## Abstract

We announce the complete genome sequence of *Klebsiella pneumoniae* strain 11978 isolated from a patient hospitalized in Turkey in 2001. The genome belongs to sequence type 14 and includes three plasmids. Notably, it presents an IncL plasmid carrying *bla*_OXA-48_, which demonstrated global success in terms of dissemination.

## ANNOUNCEMENT

*Klebsiella pneumoniae* strain 11978 harboring the *bla*_OXA-48_ gene was recovered in 2001 from the urine of a 54-year-old male patient suffering from a urinary tract infection and skin burns, hospitalized in Istanbul Faculty Hospital, Turkey (1). The strain was identified using the API 32GN system (bioMérieux, Marcy l'Etoile, France) and showed resistance not only to all β-lactams, including carbapenems, but also to many other antibiotics including aminoglycosides, chloramphenicol, rifampin, sulfonamides, nalidixic acid, and tetracycline and intermediate susceptibility to ciprofloxacin. Notably, OXA-48 was primarily identified from this isolate, being actually the first carbapenem-hydrolyzing class D ß-lactamase identified from an enterobacterial isolate (1). OXA-48 constitutes the predominant carbapenemase found in Enterobacterales in numerous countries, particularly in Europe (2, 3). It is frequently detected not only in nosocomial *K. pneumoniae* isolates but also in community-acquired *Escherichia coli* isolates (4, 5).

In this study, we report the full genome sequence of the *K. pneumoniae* strain 11978 (1). The strain was stored at −80°C and cultivated in UriSelect 4 at 37°C for 20 hours (Bio-Rad, Marnes-la-Coquette, France). Genomic DNA was freshly extracted using the DNA Clean and Concentrator kit (Zymo Research, Irvine, USA). Subsequently, the extracted genomic DNA was utilized for both short-read and long-read sequencing techniques employing Illumina (Illumina Inc., San Diego, CA, USA) and Nanopore technologies (Oxford Nanopore Technologies, Oxford, UK), respectively.

For short-read sequencing, paired-end libraries were prepared using the Nextera sample preparation kit and sequenced with an Illumina MiSeq platform generating 2 × 150 bp paired-end reads. No fragmentation of DNA was performed for preparing the long-read libraries, which were built starting from total DNA. Long-read sequencing libraries were generated using the one-dimensional chemistry ligation sequencing kit (SQK-LSK109) and sequenced using a MinION Mk1C using a R9.4.1 Flongle flow cell (FLO-FLG001). Quality control of short and long reads was performed using FastQC v0.12.1 (6). Subsequently, long reads were trimmed using Canu v2.2 (7), and then trimmed long reads and short-read sequences were assembled using UniCycler v0.5.0 (8) to generate hybrid assemblies. The circular genome was rotated to the dnaA gene. The complete circular chromosomal genome and plasmids were deposited in the National Center for Biotechnology Information’s (NCBI) Sequence Read Archive under BioProject no. PRJNA1077593. Annotation of the hybrid genome was performed using Prokka v1.14.6 (9). A total of 5,476 genes were predicted after annotation. Sequence type, species, acquired resistance, and virulence genes were detected using, respectively, MLST version 2.0 (https://www.genomicepidemiology.org/), KmerFinder v3.2 (10) , Abricate v0.3 (using both NCBI and Resfinder (CGE) databases) (11), and Kleborate tool v0.2.0 (12).

After annotation, four contigs were identified as complete circular sequences, corresponding to the chromosomal genome and three plasmids. The *bla*_OXA-48_ gene was located on the 61,881 bp IncL plasmid named pOXA-48a, which is now defined as an epidemic plasmid having a high conjugation frequency rate (13, 14). Additionally, two other plasmids were identified: a 391 kb IncHIA and a 105 kb IncFIB types, both carrying various antibiotic resistance genes (Table 1).

**TABLE 1:** Genome sequencing statistics, genome sequencing content, and antimicrobial susceptibility profile of the *K. pneumoniae* strain 11978^*a*^^,^ ^*c*^^,*d*^

Quality parameters	Illumina (short-reads)	Nanopore (long-reads)	Hybrid
Estimated bases	345,1 Mb	845,4 Mb	-
Coverage of sequencing	60 x	148 x	-
Reads generated	227,077	68,740	-
N50	190,297 bp	28,230 bp	-
L50	10	1	-
Contigs	108	4	4
Coverage contigs	32 x	40 x	34 x
Largest contig	572,842 bp	5,329,806 bp	5,297,177 bp
Total length	5,744,065 bp	5,993,371 bp	5,855,561 bp
GC content	56.68%	56.49%	56.63%

Hybrid assembled genome				
Contigs	Contig 1 (chr)	Contig 2	Contig 3	Contig 4
Accession number (size)	CP145598.1 (5,297,177 bp)	PP376100.1 (390,996 bp)	PP376101.1 (105,507 bp)	JN626286.1 (61,881 bp)
Incompatibility family	-	IncHIA	IncFIB(pQil)	IncL
Sequence type	ST14	-	-	-
Wzi type	Wzi16	-	-	-
K-locus	KL16	-	-	-
K-type	K16	-	-	-
O-locus	O1/O2v1	-	-	-
O-type	O1	-	-	-
Antibiotic resistance genes	*fosA and SHV-28*	*aac(6’)-lb, ant(2’’)-Ia,* *ant(3’’)-Ia, aph(3’’)-Ib,* *aph(6)-Id, catA1,* *mcr-9.1, OXA-47,* and *sul1^b^*	TEM-1, OXA-9, and SHV-12	OXA-48
Virulence genes	*mrkABCDFHIJ, kfuABC, and iutA*	-	-	-

Antimicrobial susceptibility	BMD - MICs (mg/L)	EUCAST interpretation
Amoxicillin	>512	R
Amoxicillin–clavulanic acid	>512	R
Ticarcillin	>512	R
Ticarcillin–clavulanic acid	>512	R
Piperacillin	>512	R
Piperacillin–tazobactam	512	R
Cephalothin	>512	R
Cefuroxime	256	R
Cefoxitin	128	R
Ceftazidime	512	R
Ceftazidime–clavulanic acid	256	R
Ceftazidime–avibactam	1	S
Cefotaxime	64	R
Cefepime	32	R
Cefepime–taniborbactam	0.5	NA
Cefepime–zidebactam	≤0.125	NA
Cefepime–enmetazobactam	16	NA
Cefpirome	128	NA
Moxalactam	64	NA
Aztreonam	512	R
Aztreonam–avibactam	1	NA
Ceftolozane–tazobactam	2	S
Meropenem	64	R
Meropenem–vaborbactam	32	R
Imipenem	64	R
Imipenem–relebactam	32	R
Colistin	0.5	S
Cefiderocol	1	S

^*a*^ Chr, chromosome; BMD, broth microdilution; MIC, minimal inhibitory concentration; NA, not applicable (breakpoint not available yet).

^*b*^ There are two copies of the gene *sul1* onto the IncHIA plasmid.

^*c*^ Broth microdilution was performed to access susceptibility profile of the strain against old and newly developed antibiotics, following the EUCAST guidelines.

^*d*^ ''-'', None.

## Data Availability

BioProject ID: PRJNA1077593. BioSample ID: SAMN39976604. Raw sequences: SRR27998988 and SRR27998987. Chromosome: CP145598.1, IncHIA plasmid: PP376100.1, IncFIB(pQil) plasmid: PP376101.1, IncL plasmid: JN626286.1 (formerly published).
